# Single-Step Production and Self-Assembly of Magnetic Nanostructures for Magneto-Responsive Soft Films

**DOI:** 10.1021/acsami.5c00992

**Published:** 2025-03-27

**Authors:** Mehran Sedrpooshan, Pierfrancesco Maltoni, Davide Peddis, Adam M. Burke, Maria E. Messing, Rasmus Westerström

**Affiliations:** † NanoLund, Lund University, 118, 221 00 Lund, Sweden; ‡ Synchrotron Radiation Research, Lund University, 118, 221 00 Lund, Sweden; § Department of Chemistry and Industrial Chemistry & INSTM RU, nM2-Lab, University of Genova, 16146 Genova, Italy; ∥ Institute of Structure of Matter, National Research Council (CNR), nM2-Lab, Via Salaria km 29.300, Monterotondo Scalo, 00015 Rome, Italy; ⊥ Solid State Physics, Lund University, 118, 221 00 Lund, Sweden

**Keywords:** self-assembly, magnetic nanoparticles, magnetically responsive, magnetic actuation, magnetic anisotropy, scalable production

## Abstract

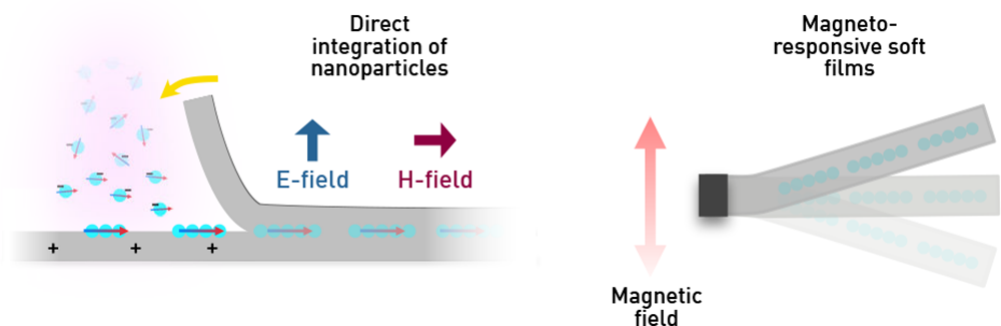

Magneto-responsive soft films constitute a fascinating class of smart materials and devices capable of performing various tasks, such as micromanipulation or transport, noninvasive surgery, and sensing. These components are fabricated by incorporating magnetic materials into flexible substrates. In this context, arranging magnetic particles into elongated chains exhibiting shape anisotropy has shown great potential. Here, we introduce a novel technique for fabricating magnetically responsive films using continuous single-step production and self-assembly of magnetic nanoparticles from a carrier gas at atmospheric pressure into anisotropic magnetic structures directly onto flexible polymer layers. We show that the resulting magnetic soft films exhibit significant residual magnetization and a large response to external magnetic fields. Furthermore, we investigate the magnetic properties of the nanoparticle assemblies and show that interparticle interactions play a critical role in determining the final magnetic properties of the nanostructures. Moreover, we provide experimental evidence that fusing the nanoparticles via post-annealing results in a transition from magnetostatic to exchange interactions with an ≈50% increase in the coercivity.

## Introduction

Magneto-responsive soft films are the active components in magnetic actuators with applications in soft robotics,^1,2^ minimally invasive diagnosis and surgical devices,^3,4^ micropumps and valves,^5,6^ metamaterials,^7,8^ and shape-morphing structures.^9,10^ Soft magneto-responsive components for actuation purposes are composed of elements fabricated by integrating magnetic materials, such as nanoparticles (NPs), into a polymer layer. In particular, the self-assembly of NPs for constructing larger structures has displayed significant potential.^9,11−16^ Specifically, arranging magnetic NPs into nanochains (NCs) has gained enormous attention for implementation in micro- and nanoactuators.^17−24^ This is due to the anisotropic properties and the high remanent magnetization of these structures resulting in an improved response to magnetic fields as the magnetic force, , and torque, , rely on the directionality and magnitude of the magnetic moment *m*. Furthermore, the nanoscale dimension of these structures allows for miniaturization of the actuators beyond what is achievable using micron-sized particles.^25^ Moreover, the potential of these versatile structures extends beyond these particular fields, as they have showcased applicability across various disciplines such as in magnetically responsive optical components,^26^ nanomedicine,^27−31^ memory and sensing devices,^32−35^ electromagnetic shielding,^36,37^ plasmonics,^38−41^ and catalysis.^42,43^

Several approaches have been developed to tune the nanoscale interactions to form one-dimensional (1D) magnetic structures on substrates or in polymer matrices. Self-assembly of colloidal NPs into 1D structures with the aid of external magnetic fields is the most common way to produce such structures on substrates or inside polymers.^16,20,24,44−46^ This approach provides simplicity and cost-efficiency. However, due to the multistep procedure and the high probability of particle agglomeration, 1D structures are mostly formed with uneven distribution, even at medium particle loading, which often leads to a deterioration of magnetic properties.^16,44−46^

The recent development of gas-phase (aerosol)-based self-assembly techniques has brought forward innovative alternatives. An important characteristic of gas-phase methods is their ability to continuously produce materials and directly integrate them onto selected substrates, which makes them advantageous for scalable production.^47−49^ These methods are based on NPs generated via physical methods in a gas and self-assembled under magnetic fields,^43,50^ electric fields,^51−53^ or the synergy of both fields to design the desired structures.^35,54^ Additionally, direct-writing techniques such as aerosol jet printing, based on aerosol impaction, have shown remarkable potential in the fabrication of flexible electronics and soft robots,^55−57^ although they are not suitable for magnetic actuation due to the utilization of sub-10 nm superparamagnetic particles, which fail to produce an effective magnetic moment and thus torque.

Here, we introduce a novel aerosol-based approach for the single-step production and self-assembly of highly anisotropic nanostructures with significant remanent magnetization onto polymer films to produce magnetically responsive components for actuation. This method employs an aerosol technique based on spark ablation for continuously generating charged magnetic NPs in a carrier gas. The NPs are subsequently attracted to a soft polymer layer by using an electric field and directly self-assembled along the direction of an applied magnetic field when reaching the surface. Unrestricted by molds and multistep procedures, the method has the potential to realize a continuous and scalable fabrication of magnetic soft films, which, to the best of our knowledge, is difficult to achieve using current multistep chemical-based techniques. Furthermore, we present a detailed magnetic characterization of the magneto-responsive films and demonstrate how the magnetic properties evolve as the assembly of NPs transforms from random to ordered NCs, and further change when these chains are transformed into fused NCs (f-NCs) by post-annealing. A detailed study is carried out by combining magnetometry analysis techniques, including first-order reversal curves (FORC) and remanence δ*m*-plots, to elucidate the correlation between morphology and magnetic interactions in particle assembly systems, which are essential for optimizing the functionality of the films.

## Results and Discussion

### Direct Integration and Fabrication of Magneto-Responsive Soft films

**Figure 1:**
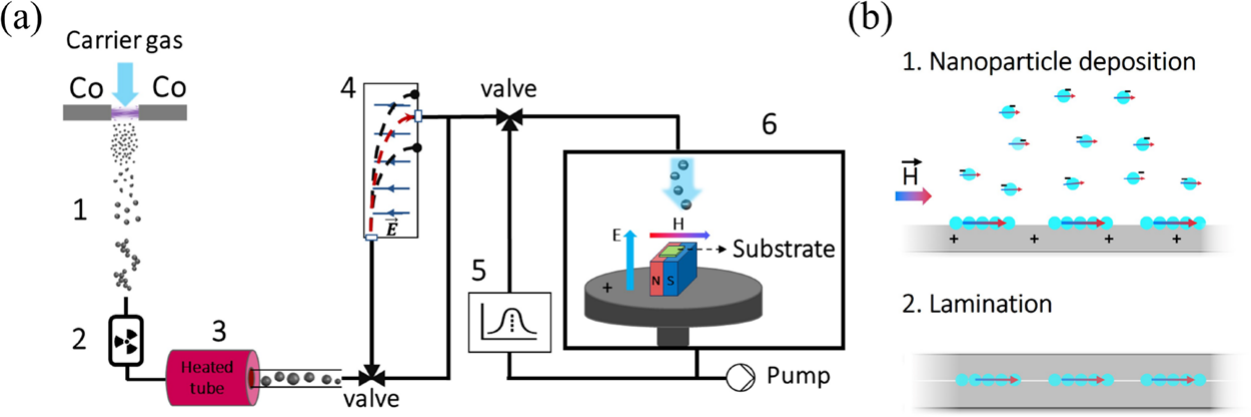
(a) Schematic of the NP formation from spark ablation of Co electrodes and self-assembly onto a polymer thin film: 1, formation of irregular Co agglomerates from the atomic cloud; 2, acquiring a known charge distribution in a radioactive ^63^Ni charger; 3, reshaping the materials into single-crystalline particles by heating at 1473 K; 4, size selection based on the electrical mobility; 5, online particle detection and analysis; and 6, deposition and self-assembly under electric and magnetic fields. (b) Direct integration of NCs onto a thin polymer layer of choice and fabrication of magnetic films by lamination.

**Figure 2:**
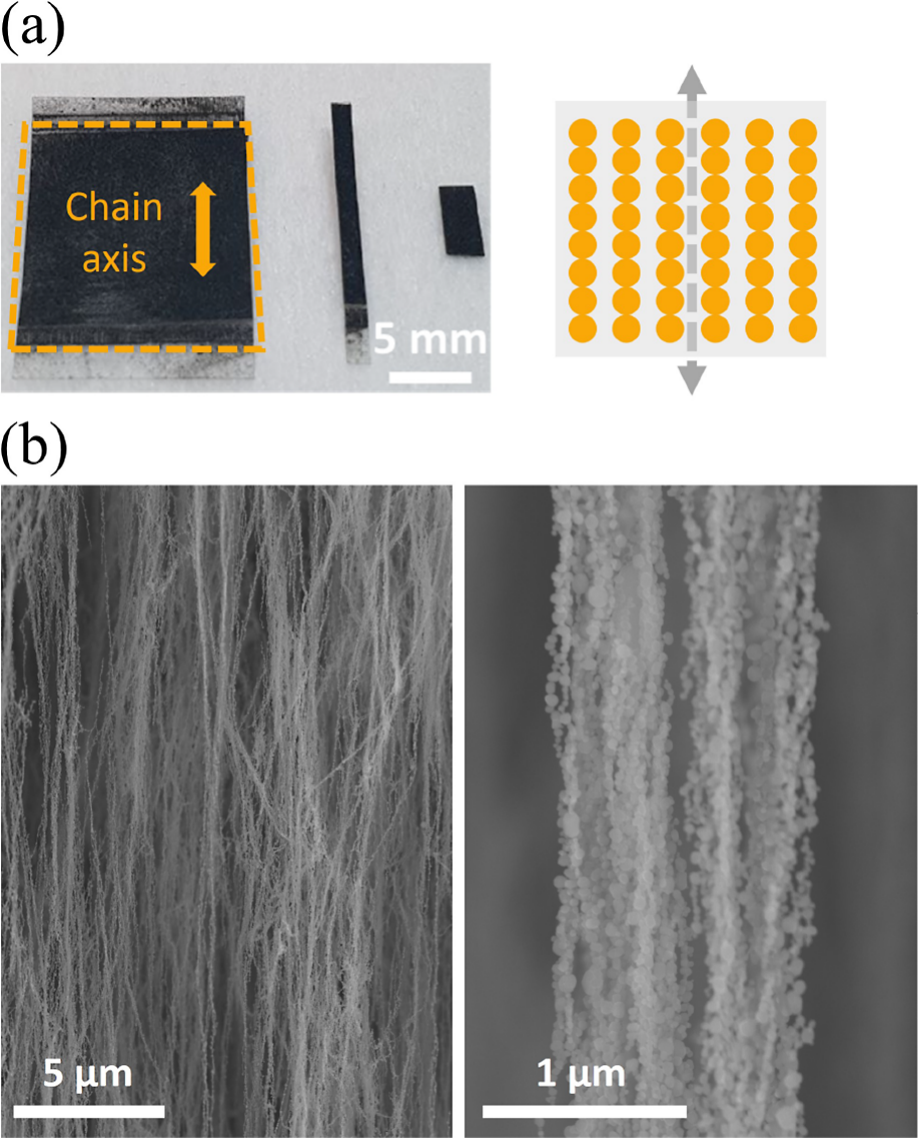
(a) Image of the magnetic soft film P2 produced by directly integrating Co NCs onto polyolefin using the approach in Figure 1 and a schematic depicting the chain orientation (axis) in the polymer layers. (b) SEM micrograph of a representative high-coverage Co self-assembled structure, showing the formation of elongated structures composed of large bundles of NCs oriented along the in-plane magnetic field applied during the particle deposition, as schematically illustrated in (a). The deposition was performed on a Si wafer to facilitate SEM imaging.

**Figure 3:**
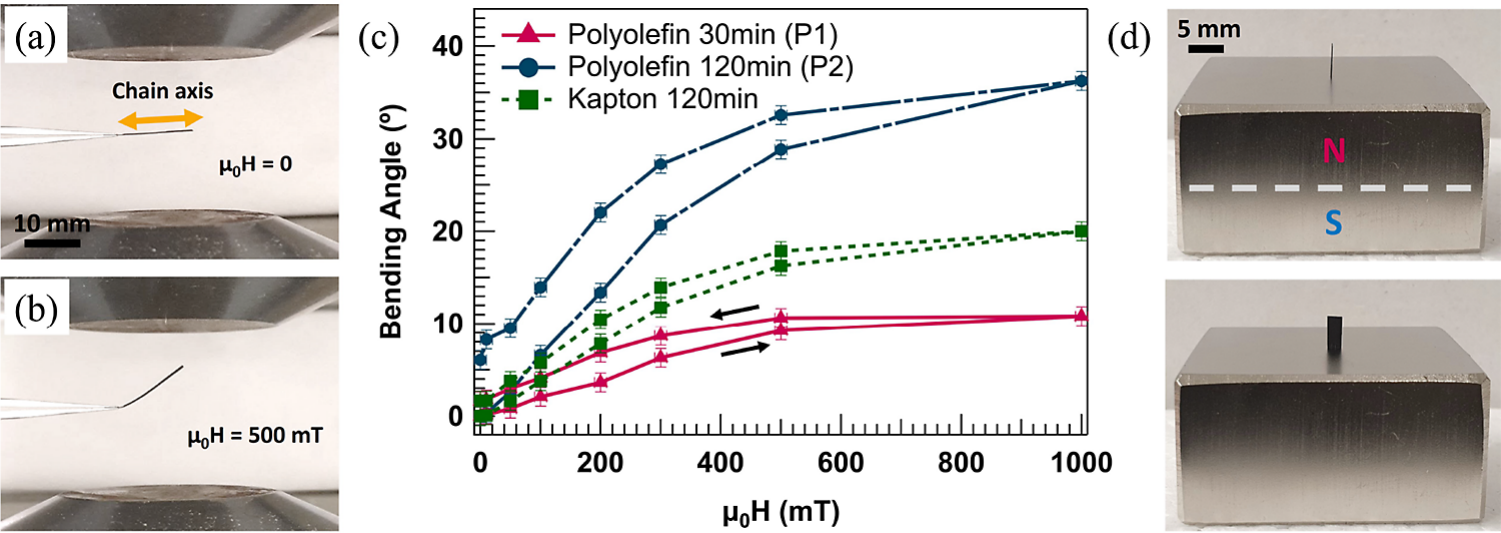
A 12.5 × 1.6 mm rectangular film cut out from P2 placed between the poles of an electromagnet at (a) 0 and (b) 500 mT fields. (c) Quantitative analysis of the bending angle per magnetic field for polyolefin and Kapton samples. (d) A 2 × 5 mm film from P2, standing vertically on a permanent magnet aligned with the stray field.

Fabricating the magnetically responsive soft films starts with generating electrically charged Co NPs suspended in a carrier gas using spark ablation,^35^ as schematically illustrated in Figure 1a and detailed in the “Experimental Section” section. The as-produced charged NPs are attracted to a flexible substrate of choice using an electric field. The substrate, here a soft polymer, is placed on a permanent magnet during the deposition, and as the NPs arrive at the surface, they self-assemble forming 1D NCs along the direction of the applied magnetic field.^35,54^ Finally, an additional polymer layer is applied to laminate and secure the NCs at the interface, as depicted in Figure 1b. Figure 2 shows SEM images of a representative high-coverage sample required for effective actuation, showing the formation of elongated structures along the external magnetic field. Here, commercially available 85 μm thick polyolefin and 70 μm thick Kapton tapes are used as substrates, resulting in magnetic films with total thicknesses of ≈170 and 140 μm after lamination. To investigate the response to external magnetic fields, two polyolefin films with dimensions ≈13 × 30 mm are produced with different particle coverages denoted P1 and P2 obtained after 30 and 120 min deposition times, resulting in magnetic layers with thicknesses of about 200 and 800 nm, respectively. The magnetic films are then cut into rectangular-shaped pieces with the chain axis oriented parallel to the longer side of the resulting film (Figure 2). A third sample with a 120 min deposition onto Kapton tape is also fabricated and investigated to demonstrate the flexibility of the technique in terms of integrating magnetic nanostructures on different polymers. Figure 3a,b displays images of a 12.5 × 1.6 mm piece of P2, under 0 and 500 mT fields. The images show that as the field is turned on, the film bends, orienting itself toward the direction of the applied magnetic field according to the magnetic torque, . The bending angles of P1 and P2 per magnetic field are plotted in Figure 3c. The results show that the bending of both samples increases nonlinearly with increasing magnetic field, with a hysteretic behavior and offset in the zero-field angle when reversing the field due to the mechanical hysteresis of the polymer.^58^ A comparison of P1 and P2 shows that P2 with a higher amount of materials exhibits an enhanced response to the external field, which is attributed to the higher magnetic moment of the film, resulting in a considerable bending angle of 36 ± 1° at 1 T. The measurements are repeated for the sample prepared using Kapton tape and are presented in Figure 3c. Compared with P2, a smaller bending angle is obtained, which can be attributed to the higher Young’s Modulus of Kapton compared to polyolefin, demonstrating the impact of the mechanical properties of polymer layer on the actuation performance. The uniaxial anisotropy of the magnetic films and their significant response to external fields are further demonstrated in Figure 3d (Video provided in the SI), where a 2 × 5 mm piece cut from P2 is vertically freestanding with its easy axis along the stray field of a NdFeB permanent magnet.

**Figure 4:**
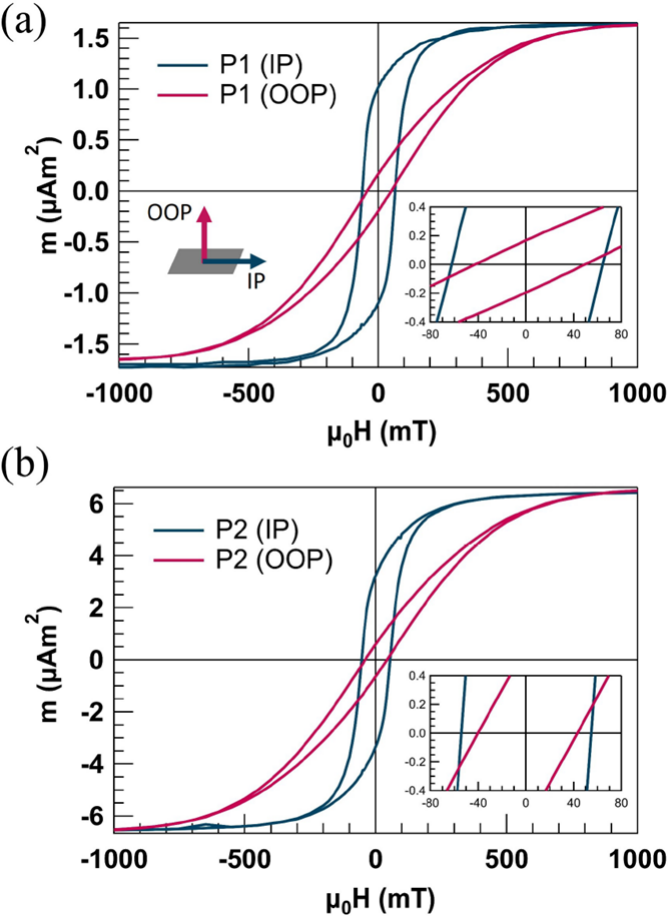
In-plane (IP) and out-of-plane (OOP) hysteresis loops of (a) P1 with *M*_r_/*M*_s_ = 0.65 and *H*_c_ = 63.3 mT and (b) P2 with *M*_r_/*M*_s_ = 0.50 and *H*_c_ = 54.3 mT (sample dimensions: 2 × 5 mm). The insets show magnified views of the regions around the coercive field. The IP measurement is performed along the chain axis.

Hysteresis loops of pieces cut out from P1 and P2 are measured and presented in Figure 4a,b, and the extracted magnetic parameters are presented in Table 1. Saturation magnetic moments of 1.7 and 6.5 μAm^2^ obtained from P1 and P2 represent an increase of about 3.85 for deposition times four times longer (30 min for P1 and 120 min for P2). This observation indicates that the deposition times can control the particle loading and thereby tune the magnetic moments and the response of the films to external fields, as seen in Figure 3c. The hysteresis loops also reveal considerable residual magnetization and anisotropy in the magnetic films, essential for effective actuation. However, as the amount of deposited material and magnetic moment of the films increases, the remanent magnetization *M*_r_/*M*_s_ decreases from 0.65 in P1 to 0.50 in P2, accompanied by a 14% reduction in the coercivity, μ_0_*H*_c_. These observations suggest that interchain interactions are critical in determining the resulting magnetic properties.

**Table 1:** Magnetic Parameters Extracted from the Hysteresis Loops Recorded at 300 K along the Chain Axis in Figures 4 and 5 (≤ ±2.5% Uncertainty)

sample	μ_0_*H*_c_ (mT)	*M*_r_/*M*_s_	μ_0_*H*_s_ (mT)	μ_0_*H*_k_ (mT)	*M*_s_ (μAm^2^)
P1	63.3	0.65	350	227	1.69
P2	54.3	0.50	450	245	6.51
S1	91.2	0.38	750	364	0.088
S2	89.8	0.74	300	263	0.088
S3	131.9	0.75	425	328	0.087

### Magnetic Response of NP Assemblies

**Figure 5:**
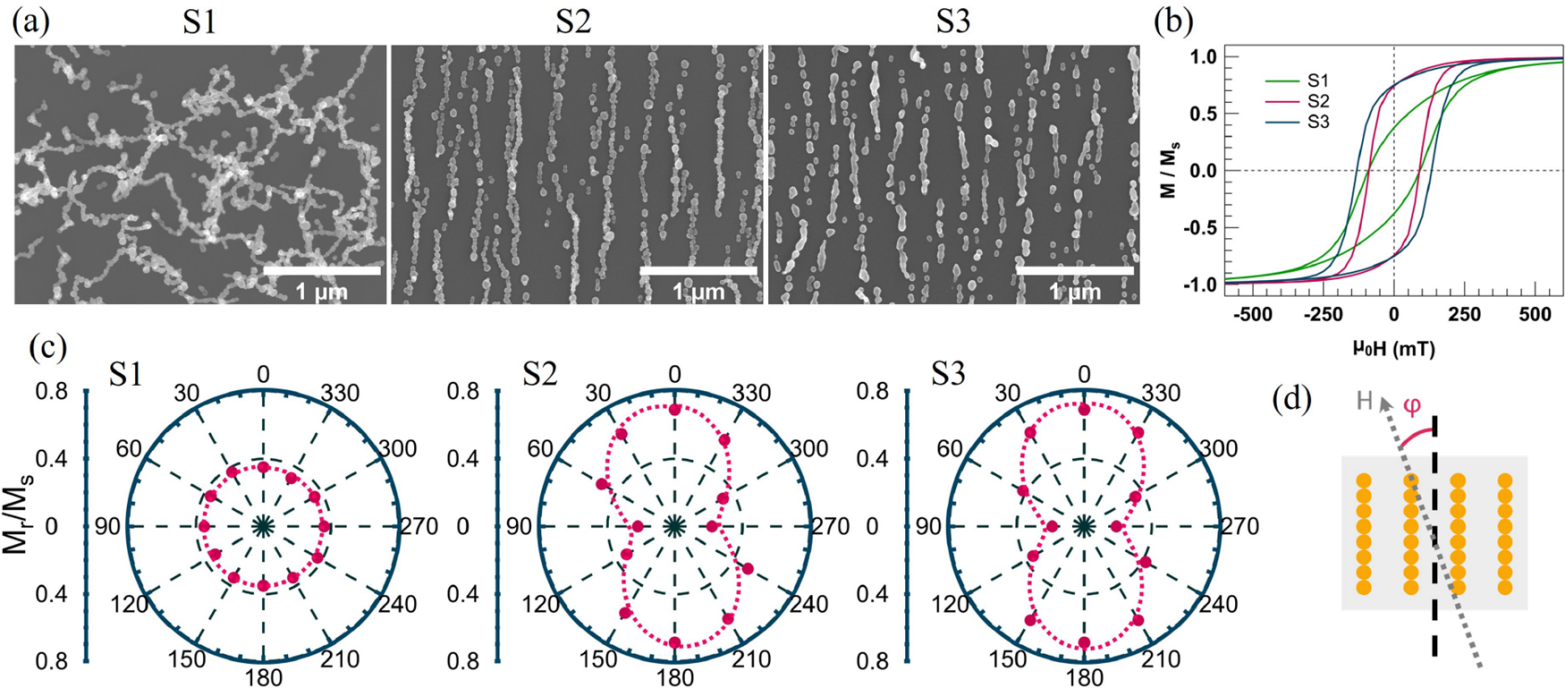
(a) SEM images of random NCs (S1), parallel NCs (S2), and parallel f-NCs (S3). (b) Magnetic hysteresis loops of S1, S2, and S3, measured at φ = 0 and 300 K. (c) Remanent magnetization per azimuthal angle demonstrating the isotropic characteristic of S1 and the uniaxial anisotropic behavior of S2 and S3. (d) Measurement geometry.

Here, we explore the magnetic properties of different Co NP assemblies to determine the influence of morphology on anisotropy, coercivity, and remanent magnetization, which are the key parameters for efficient magnetic actuation. To this end, we investigated three samples: S1, prepared without an applied magnetic field to form random particle assemblies; S2, prepared with an in-plane magnetic field to fabricate parallel NCs; and S3, f-NCs formed by post-annealing parallel chains (Figure 5). These samples are produced with significantly lower coverage to have well-defined systems for correlating the morphology of the NP assemblies with changes in the magnetic properties. Structural characterization of the Co NPs and NCs has been reported elsewhere and shows that the particles predominately have a single-crystalline *fcc* structure and self-assemble with random crystal orientations along the chains.^35^

Figure 5b shows hysteresis loops at 300 K obtained from S1 to S3, with the measurement field applied parallel to the long axis of the structures. The magnetic parameters extracted from the loops are summarized in Table 1, showing clear differences between the three systems. Forming parallel NCs leads to a significant increase in the remanent magnetization of around ≈100% compared to the randomly oriented and interconnected NCs in S1. Furthermore, f-NCs fabricated by post-annealing NCs results in a 48% increase in the coercivity. The coercivity enhancement can be explained by the establishment of direct exchange interactions and possible alignment of the crystal lattice of particles by post-annealing.^35^ The magnetic anisotropy of the structures is studied by plotting the remanent magnetization from hysteresis loops measured with the applied field oriented along a sequence of azimuthal angles. The resulting azimuthal angle dependence of the remanent magnetization in Figure 5c reveals an isotropic magnetic response for S1 and a uniaxial anisotropy for S2 and S3. The observed anisotropic response from S2 and S3 with a magnetic easy axis parallel to the long axis of the structures is a consequence of a considerable shape anisotropy induced by the elongated shape of the structures. As a result, the remanent magnetization is about three times larger when measured parallel (0°) rather than perpendicular (90°) to chains and wires. Comparing the extracted parameters from S2, P1, and P2 in Table 1 confirms that decreasing the coverage and thereby lowering interchain interactions lead to an increase in both the remanent magnetization and coercivity.

### Magnetic Interactions in the NP Assemblies

**Figure 6:**
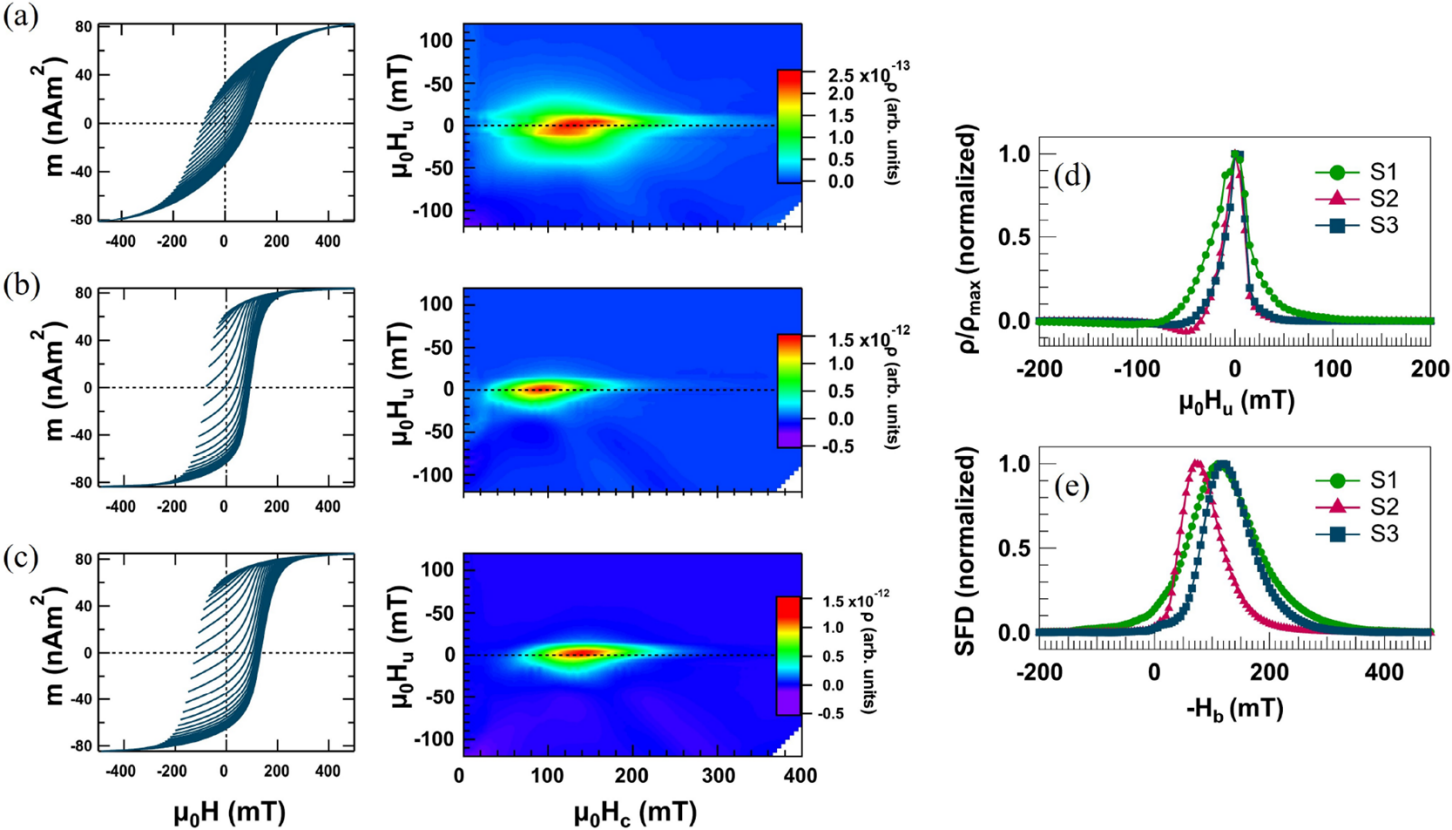
Sets of recoil loops and FORC diagrams of (a) S1, (b) S2, and (c) S3 showing the evolution of magnetic interaction uniformity and coercivity distribution as particles are arranged into parallel NCs and then post-annealed to form f-NCs. (d) Vertical projection of the FORC diagrams at the peak positions. (e) SFD curves of the three samples.

More advanced magnetic measurement protocols, such as FORC and remanence δ*m*-plots, are performed to gain insights into the magnetic interactions that determine the response of the NP assemblies to external magnetic fields. FORC measurements unveil the distribution of interactions and coercivity fields, and remanence δ*m*-plots elucidate the extent of interparticle interactions in the samples, distinguishing between possible dipolar and exchange interactions. Figure 6a–c shows the FORC minor loops and the corresponding diagrams for S1, S2, and S3. In a FORC diagram, closed contours are typically considered as the fingerprints of single-domain states, while open contours are indicative of multidomain states.^59^Figure 6a–c exhibits closed counters, suggesting a single-domain state. Furthermore, all three samples exhibit a single peak in their FORC diagrams, indicating a strongly interacting regime with uniform magnetic reversal.^59^ However, the positions of the peaks and the distributions along the horizontal and vertical axis differ. The vertical axis represents the interaction field *H*_u_, and comparing the projections of the FORC diagram at the peak positions for the three samples displayed in Figure 6d, a relatively broad distribution is observed for S1. The widening along *H*_u_ in S1 can be attributed to significant variations in the local interactions stemming from bundles and clusters with arbitrary shapes and sizes and irregular positioning. This morphological randomness in S1 also contributes to variations in switching fields, leading to broadening along *H*_c_ as each structure encounters a distinct reversal field depending upon the particle shape, orientation, and distribution, resulting in different *H*_c_ values. In contrast, the *H*_u_ and *H*_c_ distributions from the parallel NCs and f-NCs in S2 and S3 are significantly narrower, signifying more uniform interactions due to the increased regularity of the shapes, sizes, and distances between the structures. The overall similarities of the FORC diagrams of S2 and S3 indicate that post-annealing the NPs to form f-NCs does not substantially alter the interchain/wire interactions. However, the position of the S3 peak has moved to larger *H*_c_ values along the horizontal axis, consistent with the increased coercivity observed in the magnetization curves displayed in Figure 5b. Additionally, the switching field distribution (SFD) is obtained by integrating the projection of the FORC heat maps onto the *H*_b_ axis (for details, see the Experimental Section). Comparing the SFD curves in Figure 6e, S1 exhibits the widest SFD among the samples, indicating the high randomness of the reversals and interactions in this particle configuration. Moreover, forming parallel NCs moves the *H*_c_ distribution to lower values, which can be attributed to the more compact morphology and stronger interactions between parallel NCs in S2 compared to S3, which increases the dipolar interactions.^24^ The shift in the switching fields to higher values in S3 compared to S2 is also reflected in the SFD plot. These observations indicate that although the formation of parallel NCs increases the remanent magnetization in S2 compared to S1, chain alignment lowers the coercivity and SFD. Consequently, magneto-responsive films made from parallel NCs demagnetize under weaker external fields, which could potentially reduce their performance.

**Figure 7:**
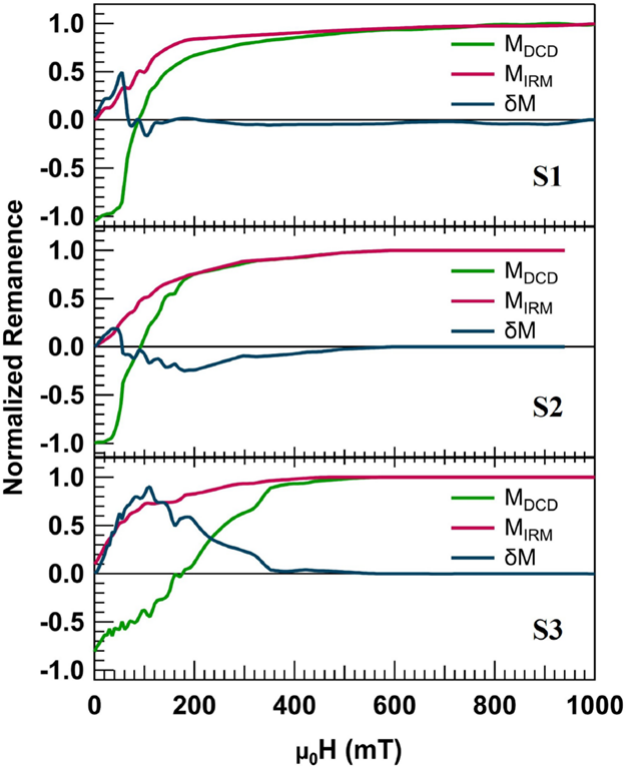
DCD and IRM remanence plots together with the δ*m*-plots of S1, S2, and S3.

To gain insights into the magnetic interactions at play, we performed remanence δ*m*-plot analysis. Figure 7 shows the normalized isothermal remanence magnetization (IRM) and direct current demagnetization (DCD) curves. Note that *m*_IRM_(*H*) starts from the demagnetized state, and the remanent magnetization is measured for each positive applied magnetic field up to 1 T (which was enough to overcome the anisotropy field), while *m*_DCD_(*H*) starts from the negative saturated state (μ_0_*H* = −1 T) and the remanent magnetization is measured for each applied positive field up to 1 T. For the ideal case of noninteracting NPs and NCs, the two remanence curves follow the Wohlfarth model, *m*_DCD_(*H*) = 1 – 2*m*_IRM_(*H*). However, in a system of interacting particles, the δ*m*-plot shows a deviation, which can be represented by the equation δ*m* = *m*_DCD_(*H*) – (1 – 2*m*_IRM_(*H*)).^60,61^ A positive peak in this curve is typically ascribed to the dominance of exchange interactions in the sample, while a negative peak shows the presence of magnetostatic interactions.^62^

Variations in the DCD and IRM remanent magnetization and the final δ*m*-plots are shown in Figure 7. The δ*m* curves from S1 and S2 show positive peaks at small fields, which is due to the blocking of the particles at room temperature, making the demagnetization process challenging and thus introducing artifacts in the lower applied fields (i.e., leaving the system with positive remanent magnetization). However, the two samples show negative peaks with different magnitudes around their coercive fields, showing the existence of dipolar interactions over a broader range of switching fields in these two samples. S2 exhibits a larger peak, demonstrating dipolar interactions between NCs, which can be attributed to the more compact morphology and shorter interchain distances also suggested by the lower coercivity values of this sample in the FORC diagram in Figure 6b. Comparing S2 and S3, the negative peak in S3 diminishes in intensity while the positive peak considerably increases, revealing the appearance of strong direct exchange interactions when forming f-NCs by post-annealing.^35^ This can be understood by the fusing of the particles at the interfaces, which brings the magnetic interactions to the atomic scale, enabling exchange interactions between particles and enhancing the coercivity of f-NCs. Although the elevated temperatures needed to fuse the NPs are incompatible with the polymers used in this work, embedding f-NCs with enhanced coercivity can be envisioned by the use of soft substrates that can sustain elevated temperatures.

## Conclusions and Outlook

In summary, we present a novel single-step technique for generating and depositing anisotropic magnetic nanostructures into various polymer layers to create soft magneto-responsive films. This technique enables the continuous production and self-assembly of magnetic NPs onto thin polymers, followed by lamination to secure the structures at the interface. The resulting magnetic soft films showed a significant response to external magnetic fields due to the considerable remanent magnetization and anisotropy of the nanostructures. Furthermore, we demonstrate that different polymers can be used and show the possibility of controlling the response to magnetic fields through particle loading. Magnetic characterization of distinct particle assemblies reveals a single-domain state with significant shape anisotropy along the NCs and that the interparticle interactions, influenced by the coverage and morphology of structures, critically affect the final magnetic properties.

**Figure 8:**
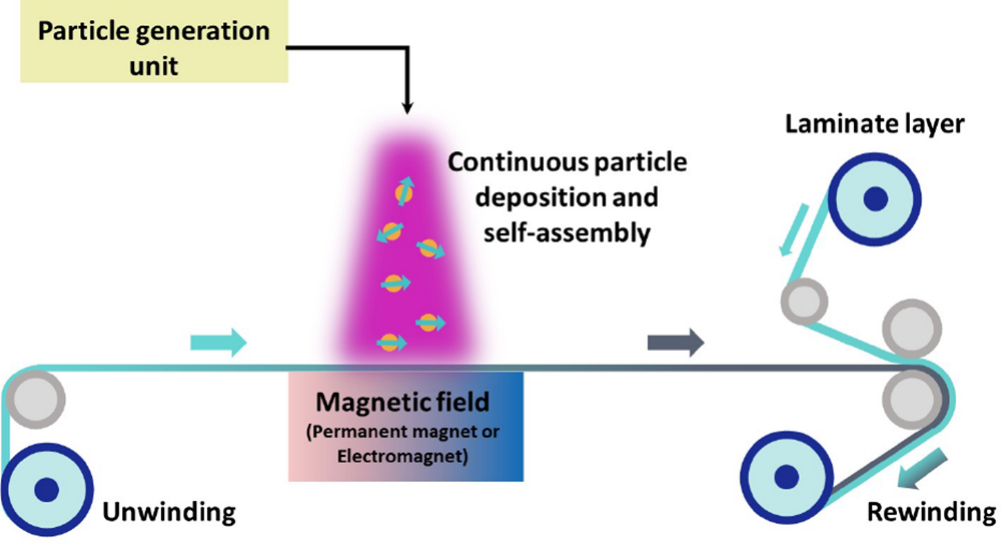
Conceptual illustration of how the presented technique could be utilized for continuous manufacturing of magnetic films by directly integrating anisotropic structures onto a soft polymer layer and securing the structures by lamination.

As an outlook of this work, inspired by the success of magnetic tape memories in large-scale production,^63^ we envision a scalable and continuous approach for producing and directly integrating magnetically responsive elements onto polymers to produce flexible magnetic films. Figure 8 shows a conceptual schematic of the approach based on a roll-to-roll system, where a pristine polymer layer is coated with the anisotropic structures and then laminated and rolled. The proposed approach could be combined with various premade polymer layers, each with specific thicknesses and properties tailored to meet the actuation requirements for particular purposes.

## Experimental Section

Spark ablation is used for generating NPs in the gas phase. The generation of Co NPs is initiated with the evaporation of materials by repetitive sparks between two high-purity metallic Co rods (5.0 mm diameter) under the flow of a carrier gas.^64^ To avoid oxidation of particles, a carrier gas composed of 95% N_2_ and 5% H_2_ (1.68 lpm) is used during the process, and the pressure of the gas is kept constant around 1015 mbar.^35,65,66^ Condensation of the vapor results in sub-10 nm primary particles, which subsequently form larger irregular agglomerates through coalescence and collisions. These agglomerates are then passed to a Ni^63^ bipolar diffusion charger to find a known charge distribution. After that, the agglomerates are passed through a tube furnace, where they are compacted into faceted NPs at 1473 K and then size-selected (*D* = 40 nm) based on their electrical mobility using a differential mobility analyzer. The deposition of particles is performed in an electrostatic precipitator,^67^ and for the deposition under a magnetic field, substrates are placed on a permanent magnet with an in-plane field of ≈ 300 mT. For depositing a desired amount of particles on substrates, the particle concentration in the gas is monitored online with a TSI Electrometer Model 3068B, and the deposition time is calculated.^68^ Subsequently, for transforming chains of particles into f-NCs, rapid thermal annealing is carried out, using an RTP-1200–100 from UniTemp GmbH, at 773 K for 2 min under a forming gas of 95% N_2_ and 5% H_2_. Scanning electron microscopy is performed using a Hitachi-SU8010 cold field-emission scanning electron microscope.

In the high-coverage samples fabricated for actuation, the size distribution of particles around 40 nm is widened in order to include more particles and obtain a high deposition yield. In this case, the deposition is carried out on polyolefin tape (Semiconductor Equipment Corporation, DU-300, with an acrylic adhesive layer) and Kapton tape (Hi-Bond Tapes Ltd., polyimide HB830, with a silicone adhesive layer). Pieces of the samples with small variations in size are then manually cut out for bending angle and hysteresis loop characterizations. Bending tests are performed by clamping magnetic films from one end and placing them between the poles of an electromagnet. Subsequently, the bending angle for each field is obtained through image analysis, defined as the angle between the horizon and the line connecting the tip of the clamp to the tip of the film.

To investigate the magnetic behavior of the self-assembled systems, a commercial vector vibrating sample magnetometer (MicroSense Model 10 VSM) equipped with a rotating electromagnet is utilized for obtaining hysteresis loops of the produced magnetic films. The same equipment is employed for the angular remanent magnetization measurements and δ*m*-plots. In the latter case, remanent magnetization of structures, produced on single-crystalline SiO_2_ substrates, was measured by means of IRM and DCD protocols.^69,70^ IRM curves are obtained starting from a demagnetized sample at μ_0_*H* = 0 T and measuring the remanence corresponding to increasing fields μ_0_*H* > 0; DCD curves are obtained in the same manner, but the measurement starts from μ_0_*H* = 0 with a sample previously being saturated at μ_0_*H* = −1 T. To study magnetic interparticle interactions, normalized IRM and DCD magnetizations can be compared by means of the Kelly equation, providing the so-called δ*m*-plot:^60,61^ δ*m* = *m*_DCD_(*H*) – (1 – 2*m*_IRM_(*H*)). Hysteresis loops and FORC measurements are obtained using an MPMS 3, Quantum Design, superconducting quantum interference device vibrating sample magnetometer (SQUID-VSM). FORC measurements are conducted by first saturating the sample and then collecting M–H curves from a reduced field of *H*_a_ to 500 mT. After each partial curve, the reduced field was further lowered by 10 mT until reaching −500 mT. FORC diagrams are obtained using FORCinel^71^ based on , with *H*_b_ being the measurement field. After the partial differentiation, the data are plotted based on , the coercive field, and , the interaction field. The anisotropy field, μ_0_*H*_k_, can be approximated to the irreversible field, μ_0_*H*_irr_ (Table 1), which is defined as the field at which the difference between the major magnetizing and demagnetizing branches of the hysteresis loop is lower than 1%.^72^

## Supporting Information Available

Response of the magnetic film shown in Figure 3d to external fields (Video S1) (MP4)
